# Process Capability Assessment and Surface Quality Monitoring in Cathodic Electrodeposition of S235JRC+N Electric-Charging Station

**DOI:** 10.3390/ma19020330

**Published:** 2026-01-14

**Authors:** Martin Piroh, Damián Peti, Patrik Fejko, Miroslav Gombár, Michal Hatala

**Affiliations:** 1 Faculty of Manufacturing Technologies with a Seat in Prešov, Technical University of Kosice, Štúrova St. 31, 080 01 Prešov, Slovakia; martin.piroh@tuke.sk (M.P.); patrik.fejko@tuke.sk (P.F.); michal.hatala@tuke.sk (M.H.); 2 Faculty of Mechanical Engineering, University of West Bohemia Pilsen, Univerzitní 8, 306 14 Plzeň, Czech Republic; gombar.mirek@gmail.com

**Keywords:** cathodic electrodeposition, coating thickness, statistical process control, process capability, ANOVA, inter-station variability, machine learning prediction, residual analysis, FMEA

## Abstract

This study presents a statistically robust quality-engineering evaluation of an industrial cathodic electrodeposition (CED) process applied to large electric-charging station components. In contrast to predominantly laboratory-scale studies, the analysis is based on 1250 thickness measurements, enabling reliable assessment of process uniformity, positional effects, and long-term stability under real production conditions. The mean coating thickness was specified at 21.84 µm with a standard deviation of 3.14 µm, fully within the specified tolerance window of 15–30 µm. One-way ANOVA revealed statistically significant but technologically small inter-station differences (F(49, 1200) = 3.49, *p* < 0.001), with an effect size of η^2^ ≈ 12.5%, indicating that most variability originates from inherent within-station common causes. Shewhart X^¯^–R–S control charts confirmed process stability, with all subgroup means and dispersions well inside the control limits and no evidence of special-cause variation. Distribution tests (χ^2^, Kolmogorov–Smirnov, Shapiro–Wilk, Anderson–Darling) detected deviations from perfect normality, primarily in the tails, attributable to the superposition of slightly heterogeneous station-specific distributions rather than fundamental non-Gaussian behaviour. Capability and performance indices were evaluated using Statistica and PalstatCAQ according to ISO 22514; the results (Cp = 0.878, Cpk = 0.808, Pp = 0.797, Ppk = 0.726) classify the process as conditionally capable, with improvement potential mainly linked to reducing positional effects and centering the mean closer to the target thickness. To complement the statistical findings, an AIAG–VDA FMEA was conducted across the entire value stream. The highest-risk failure modes—surface contamination, incorrect bath chemistry, and improper hanging—corresponded to the same mechanisms identified by SPC and ANOVA as contributors to thickness variability. Proposed corrective actions reduced RPN values by 50–62.5%, demonstrating strong potential for capability improvement. A predictive machine-learning model was implemented to estimate layer thickness and successfully reproduced the global trend while filtering process-related noise, offering a practical tool for future predictive quality control.

## 1. Introduction

Cathodic electrodeposition (CED) has become a dominant surface-coating technology in the automotive and electrical-equipment industries due to its ability to produce uniform, adherent and corrosion-resistant coatings on complex geometries under highly controlled electrochemical conditions [1,2,3]. Its industrial relevance continues to increase as manufacturers shift toward large structural assemblies, such as electric-vehicle charging stations requiring stable long-term production capability, robust surface quality and minimal variability in the applied coating layer. Despite widespread industrial usage, the underlying relationships between pretreatment conditions, deposition parameters, mechanical performance and statistical process behaviour remain insufficiently mapped, particularly when considering full-scale production environments [4,5].

A significant body of research has therefore addressed the influence of surface pretreatment, consistently demonstrating that the degreasing stage strongly affects final coating behaviour. Peti et al. [6] employed a Design of Experiment (DoE) methodology to quantify how degreasing temperature (40–80 °C), chemical concentration and immersion time influence layer thickness formation on VDA 239-100 CR4 steel [7]. Their results revealed systematic differences exceeding ±5 µm between extreme pretreatment conditions, indicating that insufficient or overly aggressive degreasing can disrupt coating coalescence, modify the nucleation behaviour on the substrate, and induce non-uniform film formation [8]. A follow-up study [9] confirmed these trends with respect to chemical concentration of Pragolod 57N, identifying that inadequate concentration reduces deposition efficiency, while excessive concentration leads to non-uniformity and increased localized defects due to intensified surface reactions. The mechanical consequences of pretreatment variability were further expanded by Fejko et al. [10], who showed on AW1050A H24 aluminium that crack initiation and propagation during bending tests vary dramatically (1–100 mm) as a function of degreasing conditions. The magnitude of this variation far exceeds the thickness fluctuations reported in experimental studies [6,9], demonstrating that mechanical loading amplifies underlying pretreatment-induced deficiencies. Altogether, these studies establish that pretreatment plays a multiplicative role—first affecting electrophoretic deposition and later governing the coating’s mechanical durability [11]. Beyond pretreatment, numerous investigations have addressed the influence of deposition parameters themselves. Rossi et al. [12] demonstrated that increasing deposition voltage enhances coating thickness and continuity on aluminium foam substrates, while simultaneously promoting localized excess build-up and entrapped microdefects. Their microstructural analysis distinguished coated, partially coated and resin-residual regions, illustrating the challenge of achieving uniform deposition on complex 3D porous structures. Zanella et al. [13] confirmed that the substrate’s electrochemical nature significantly affects the final coating structure and adhesion, showing that active steel, passive nickel and noble gold—despite identical deposition parameters—produce coatings with markedly different barrier and adhesion characteristics. Complementary studies by Almeida et al. [14] and Deflorian et al. [15] highlighted the importance of curing and polymerization regimes, showing that insufficient curing decreases adhesion and water resistance, whereas excessive curing induces internal stresses and microcrack formation [16]. These polymerization-induced phenomena were also quantified by Peti et al. [17], who revealed a non-linear relationship between polymerization time, curing temperature and crack length on CR4/CR5 steel, with prolonged curing at elevated temperatures producing cracks nearly twice as long compared to shorter curing times [18,19]. This behaviour aligns closely with the given findings, confirming that coating integrity is a combined outcome of pretreatment quality, deposition kinetics, and polymerization-induced structural evolution. The interdependence of these technological factors has also been evaluated from a microstructural perspective. Continuous research [20] has shown through SEM–EDX cross-sectional analysis that optimized pretreatment and deposition conditions not only improve compositional uniformity but also stabilize the thickness formation mechanism. The authors reported average dry-film thickness values ranging from 17.7 to 22.0 µm under optimal combinations of voltage (250–300 V), deposition time (5–7 min) and low-temperature degreasing (40–50 °C), while non-optimized conditions produced significantly thinner and spatially inconsistent layers (as low as 12.2–14.8 µm at 200 V or 80 °C degreasing). Elemental mapping across the 20–25 µm coating profile further indicated a high and uniform carbon content (≈49 wt.%), stable oxygen distribution (≈6–7 wt.%) and minimal diffusion of substrate elements such as Fe and Al toward the coating, confirming a well-formed organic matrix. Silicon-rich regions within the upper layer (≈20–24 wt.%) were homogeneously distributed, corresponding to paint additives that contribute to film continuity and barrier properties. Similarly, Skotnicki and Jędrzejczyk [21] reported significantly improved coating thickness uniformity and adhesion after chemical cleaning compared to abrasive blasting. Their measurements showed that chemically cleaned surfaces produced a highly uniform CED coating with an average thickness of 33 µm and a very low variability (s.d. = 1.62 µm), while abrasive-blasted substrates exhibited thickness values ranging from 18 to 34 µm with more than a threefold increase in standard deviation (s.d. = 6.03 µm). SEM analysis further revealed a compact, low-porosity microstructure for chemically cleaned samples, in contrast to the cracked and heterogeneous morphology observed after abrasive blasting. These differences translated into improved functional performance: chemically cleaned specimens achieved a lower and more stable friction coefficient (µ ≈ 0.13 vs. µ ≈ 0.35) and demonstrated a more homogeneous wear track profile. Although the corrosion onset time in Neutral Salt Spray (NSS) corrosion testing (72 h) was similar for both variants, the chemically cleaned surfaces showed a markedly smaller corroded area, indicating better barrier integrity and reduced defect density.

Parallel to technological research, another stream of studies emphasized the importance of statistical evaluation and process capability analysis for industrial CED processes. Peti et al. [22] demonstrated a full Computer-Aided Quality control (CAQ) implementation applied to charging-station components (U009578, U009576 and U009575), integrating Statistical Process Control (SPC) charts and capability indices according to IATF 16949:2016. Their analysis—based on approximately 30 measurements per part—showed that the process was conditionally capable (Process Capability Index Cp ≈ 2.74, 1.83, 2.45; Process Capability Index (Centered) Cpk ≈ 0.8, 2.54, 1.53, 2.16 and Process Performance Index Pp ≈ 1.95, 1.59, 1.48; Process Performance Index (Centered) Ppk ≈ 1.83, 1.33, 1.30), with primary limitations arising from mean shifts and positional variability. Later, their research [23] expanded this methodological framework by integrating mechanical testing, layer-thickness evaluation and detailed capability modelling, demonstrating that coatings fulfilling nominal specification limits (typically 18–22 µm) may still exhibit insufficient long-term capability due to distribution asymmetry, heteroscedasticity and systematic mean displacement. Their results revealed that although average thickness values remained within the required interval, the presence of non-normal tail behaviour, left-skewed distributions, and voltage-dependent shifts in film build-up led to reduced capability indices (e.g., Cpk < 1.0) during extended polymerization times. This confirmed that microstructural mechanisms, particularly polymerization-induced internal stresses and crack-length evolution, directly manifest as statistical instability over time, highlighting that specification conformity alone cannot guarantee long-term process capability. Despite these significant advancements, contemporary literature is still constrained by several fundamental limitations. Most studies employ relatively small laboratory datasets, typically ≈ 10–30 specimens conducted on simplified geometries and under highly controlled conditions. Such data do not capture real industrial influences, including operator variability, bath-chemistry drift, geometric shadowing, or hydrodynamic flow differences across large assemblies. Furthermore, none of the existing studies systematically evaluate surface segments based on industrial inspection zones (directly visible, partially visible, hidden), despite their critical importance in assessing coating uniformity and customer-facing quality. Finally, although capability analyses have been conducted, none integrate positional ANOVA, distribution analysis, and spatial segmentation into a cohesive evaluation framework, and no published study to date includes more than 1000 real-production measurements capable of representing long-term process behaviour [24].

The present study directly addresses these limitations by presenting the large-scale, station-resolved industrial assessment of cathodic electrodeposition (CED) coatings applied to full-size S235JRC+N electric charging-station enclosures. Unlike the majority of existing studies, which are typically based on small laboratory datasets and simplified geometries, this work evaluates a real production environment using a statistically robust dataset comprising 1250 coating-thickness measurements collected from 50 predefined control stations over a three-month period.

The novelty of this research lies not only in the scale of the experimental dataset, but also in the integrated analytical framework that combines spatially resolved one-way ANOVA, multi-level statistical process control, ISO 22514-7 [25]-compliant capability and performance indices, AIAG–VDA FMEA and machine-learning-based thickness prediction within a single coherent study. This integrated approach enables simultaneous evaluation of spatial variability, distributional behaviour, process stability and risk-related technological factors, which have so far been addressed only separately or under laboratory conditions in the literature. By explicitly linking station-dependent statistical variability to technological failure modes such as surface contamination, improper hanging orientation and bath-chemistry instability, the present study delivers an industrially validated and transferable framework for understanding process stability, thickness variability and surface-quality behaviour of CED coatings under real manufacturing conditions.

## 2. Materials and Methods

The experimental investigation was performed on enclosure assemblies forming part of an electric-vehicle charging station, produced from normalized non-alloy structural steel H3 S235JRC+N in accordance with EN 10025-2:2004 [26]. The material was selected based on its favorable combination of mechanical and metallurgical properties, including a balanced strength-to-ductility ratio, fine-grained ferritic–pearlitic microstructure, and verified weldability, which collectively ensure dimensional stability and low residual stress formation during manufacturing and coating. Its uniform electrical conductivity and isotropic microstructural morphology provide an optimal substrate for the deposition of cataphoretic or cathodic electrodeposition coatings.

The primary aim of the experimental methodology was to determine the stability, repeatability, and capability of the industrial CED process in the context of ensuring conformance with the stringent quality standards governing series production. The investigation emphasized the relationship between the electrochemical deposition parameters, the spatial distribution of the dry-film thickness (DFT), and the resultant corrosion-resistance potential of the protective coating system. To ensure reproducibility and quantitative validity, the procedure was established under the principles of Statistical Process Control (SPC). The DFT of the deposited layer was measured using a calibrated magnetic induction instrument—Elcometer 456C by Elcometer Ltd., Manchester, UK, operating under a metrological verification regime to maintain traceability and accuracy according to ISO 2808:2019 [27]. Measurements were acquired at 25 fixed control points on the frontal surface of each enclosure directly upon removal from the CED production line. Data collection extended over a three-month observation period, thereby enabling an evaluation of both short-term and long-term process dispersion characteristics.

The acquired datasets were statistically processed in the Palstat CAQ environment (version 2025.02.002, Palstat s.r.o., Vrchlabí, Czechia), which integrates modules for SPC, Measurement System Analysis (MSA), and Process Capability Evaluation (PCE) in compliance with ISO 22514-7:2021 [25], IATF 16949:2016 [28] and ISO 9001:2015 [29]. The platform supports automatic data import, generation of control charts, and capability-index computation (Cp, Cpk, Pp, Ppk) within the defined specification limits. Normality of the measured data was verified using the Anderson–Darling test prior to the determination of capability indices. Short-term indices (Cp, Cpk) were employed to characterize intrinsic variability under stable process conditions, whereas long-term indices (Pp, Ppk) reflected cumulative variations throughout the observation period. The control limits were established between the lower specification limit Lower tolerance boundary (LSL) of 15 µm and the Upper tolerance boundary (USL) of 30 µm, consistent with industrial standards for cathodic deposition [30].

### 2.1. Material Selection—S235JRC+N

The substrate material S235JRC+N was selected as a representative low-carbon, non-alloy structural steel for enclosure manufacturing. This material, standardized by EN 10025-2:2004 [26] is characterized by its excellent weldability, cold formability and consistent strength-to-ductility response, which collectively ensures the mechanical integrity required for post-treatment surface processes. The normalized delivery condition (denoted “+N”) ensures a refined grain structure, uniform mechanical properties across the section thickness, and reduced internal stress gradients, thereby minimizing deformation and enhancing adhesion of subsequently deposited organic or electrochemical coatings (Table 1).

The chemical composition ensures low carbon equivalence and adequate toughness, while the fine-grained normalized structure provides isotropic mechanical behaviour. Mechanical parameters specified by the standard are summarized in Table 2.

### 2.2. Cathodic Electrodeposition Process

**Figure 1:**
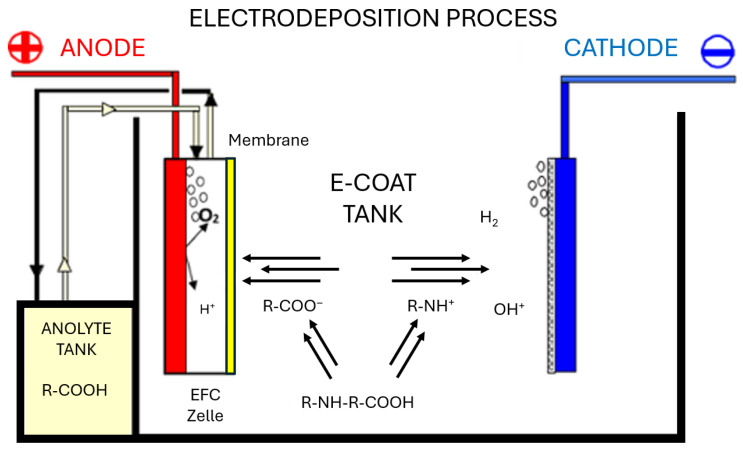
Representation of the cathodic electrodeposition process for base coatings.

Cathodic electrodeposition represents an electrochemical coating technique used extensively in the automotive and electrical industries for the formation of compact, defect-free organic layers with superior corrosion resistance. The mechanism of film formation shown in Figure 1 is governed by the balance of electrophoretic mobility, diffusion-limited boundary-layer transport, and hydration-shell dynamics of the dispersed particles. Continuous agitation of the coating bath prevents sedimentation, maintains uniform dispersion of solids, and stabilizes the deposition rate. Under controlled operational parameters (Table 3), the process ensures consistent film growth and complete coverage even on geometrically complex substrates [31].

**Figure 2:**
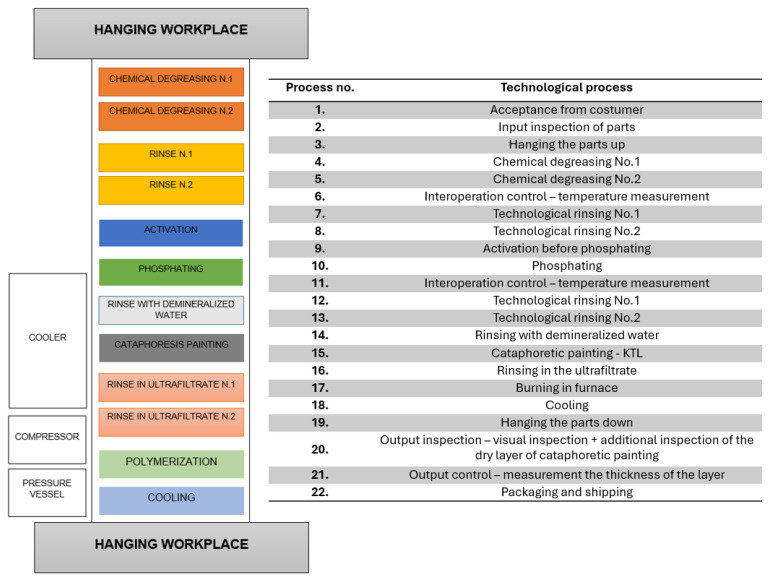
Overview of the cathodic electrodeposition (CED) line.

The process employed for the experimental campaign (Figure 2) comprised sequential operations including chemical degreasing, phosphating, ultrafiltration rinsing, electrodeposition, thermal curing, and controlled cooling. During deposition, an external DC voltage drives the migration of charged polymeric particles suspended in a water-based medium toward the cathodic substrate, where electrochemical neutralization and film coalescence occur, producing a continuous layer typically ranging between 10 and 25 µm in thickness [32,33].

Cathodic electrodeposition and pre-treatment parameters applied in this study were selected according to stable industrial production settings recommended by the coating-line supplier used in series manufacturing of KTL, s.r.o., Prešov, Slovakia. The objective of the present study was not the optimization of individual process parameters, as commonly addressed in previous investigations [6,17,20,21], but to statistically evaluate process stability, spatial variability, and capability under real production conditions. Compared with conventional coating routes, the applied CED conditions (Table 3) demonstrate superior environmental and operational performance through reduced volatile organic compound (VOC) emissions, efficient material utilization via ultrafiltration recovery, and excellent throwing power. These characteristics enable reproducible coating of recessed regions and complex geometries while maintaining mechanical integrity and aesthetic consistency, which is essential for large-scale industrial production of electric charging-station enclosures.

### 2.3. Experimental and Design of Experiment Set Up

**Figure 3:**
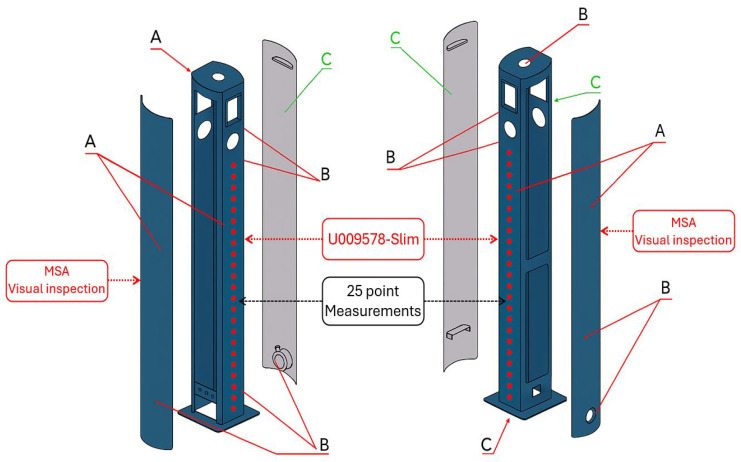
EV charging station layout with designated CED coating thickness measurement points [µm]. A—Red zone (directly visible surface): Defects or irregularities identified on these surfaces must be carefully removed by fine abrasive sanding. B—Orange zone (partially visible surface): These areas require a preventive quality control approach comparable to that applied to directly visible zones. C—Green zone (non-visible surface): Post-cataphoretic coating imperfections in these regions do not require corrective intervention.

Figure 3 illustrates the structural segmentation and inspection zones of the analyzed electric charging station components. Individual surface regions were classified according to their visibility and functional relevance in the final assembly to align inspection and evaluation procedures with industrial quality requirements. Visual inspection was performed as part of Measurement System Analysis (MSA), focusing on side panels to verify surface uniformity and detect visible defects or irregularities. Dry-film thickness measurements were conducted on the U009578-Slim component, which constitutes the primary load-bearing structure of the charging station.

To evaluate coating uniformity and process stability, a statistically robust and reproducible measurement scheme was implemented for dry-film thickness assessment on S235JRC+N electric charging station housings (U009578-Slim). Each dataset consisted of 50 measurements acquired at 25 predefined control points (*n* = 1250) distributed across the frontal surface, ensuring balanced spatial coverage and traceability (Figure 3), covering regions susceptible to electrochemical field gradients and geometric shielding effects associated with the hanging configuration. This approach ensured consistent spatial coverage across all deposition stations and enabled reliable evaluation of inter-station variability and local coating uniformity. The selection of the control points was guided by component geometry and industrially recognized high-risk areas for thickness non-uniformity. All measurement points were deliberately located on planar surface regions to ensure metrological reliability of thickness readings. Their spatial distribution was designed to represent areas influenced by geometric features of the component, electrochemical field gradients, and potential shielding effects. The longitudinal arrangement of the points along the component height enables capturing systematic thickness gradients related to bath hydrodynamics, electrode positioning, and current density distribution during cathodic electrodeposition.

Data evaluations were performed in the PalstatCAQ environment, integrating statistical quality control with FMEA-based risk assessment. This framework enabled CED process evaluation in accordance with IATF 16949:2016 and ISO 9001:2015 requirements.

### 2.4. Thickness Measurement

DFT of the cataphoretic coating applied to the S235JRC+N steel housings of the electric charging stations was measured using a digital Elcometer 456C thickness gauge (Elcometer Ltd., Manchester, UK)-serial number XH22928 with a probe specifically designed for steel substrates according to EN 2808:2019 [27]. The gauge operates in a measurement range of 0–1500 μm, with a resolution of 0.1 μm for thicknesses between 0 and 100 μm and 1 μm for 100–1500 μm. The measurement accuracy is specified as ±1–3% or ±2.5 μm, providing highly reliable readings suitable for detailed quality assessment of coating layers [34]. The Elcometer 456C complies with a wide range of international standards for coating thickness measurement, including AS 2331.1.4 [35], ASTM B 499 [36], ASTM D 1186-B [37], ISO 1461 [38], ISO 2360 [39], ISO 2808 [27], DIN 50981 [40], DIN 50984 [41], and IMO MSC.244(83) [42]. Calibration was verified using a standard calibration plate (Part number 4910, Serial number XJ06167 and an etalon thickness of 24.1 μm (Tracking number TXG90344), ensuring traceability and measurement accuracy.

### 2.5. PalstatCAQ Software

The statistical evaluation and capability analysis of the cathodic electrodeposition process were conducted using the PalstatCAQ software (version 2025.02.002) [43]. This modular computer-aided quality management platform integrates Statistical Process Control (SPC), Measurement System Analysis (MSA), and process capability assessment functionalities in compliance with ISO 22514-7:2021 and IATF 16949:2016 standards. The software enables automated import of measurement data, generation of control charts, and calculation of capability indices of both short-term (Cp, Cpk) and long-term (Pp, Ppk) process performance within predefined specification limits, ensuring full traceability of results.

The software environment enabled automated calculation of process capability indices using standard Equations (1)–(4):

(1) $$CP\;=\;\frac{USL-LSL}{6\sigma \;Short\;term}$$

(2) $$PP=\frac{USL-LSL}{6\sigma \;Long\;term}$$

(3) $$Cpk=Min\;\{\frac{USL-\mu }{3\sigma \;Short\;term};\;\frac{\mu -LSL}{3\sigma \;Short\;term}\}$$

(4) $$Ppk=Min\;\{\frac{USL-\mu }{3\sigma \;Long\;term};\;\frac{\mu -LSL}{3\sigma \;Long\;term}\}$$

where USL and LSL denote the upper and lower specification limits (15 µm and 30 µm), μ is the process mean, and σ represents the process standard deviation. The short-term indices (Cp, Cpk) reflect the intrinsic capability of the process under controlled conditions without considering long-term variability, whereas the long-term indices (Pp, Ppk) evaluate the overall stability and centering of the process over extended production periods. According to established industrial quality standards for surface coating processes, the following acceptance criteria were adopted when assessing capability performance.

According to IATF 16949:2016 and industry practice for cathodic electrodeposition processes, Cp and Cpk values ≥ 1.33 indicate a capable and statistically stable process, while Pp and Ppk values ≥ 1.50 denote robust long-term process performance (Table 4).

The capability analysis was performed in the PalstatCAQ environment, complemented by an AIAG–VDA FMEA to identify and mitigate key sources of process variability.

## 3. Results

Table 5 provides a summary of the descriptive statistics for the coating thickness dataset—the results indicate a centrally clustered thickness distribution with moderate variability relative to the specification window, reflecting stable and reproducible film formation across the evaluated deposition stations. The absence of pronounced kurtosis further suggests that extreme thickness deviations occur infrequently and are not characteristic of the process behavior.

**Figure 4:**
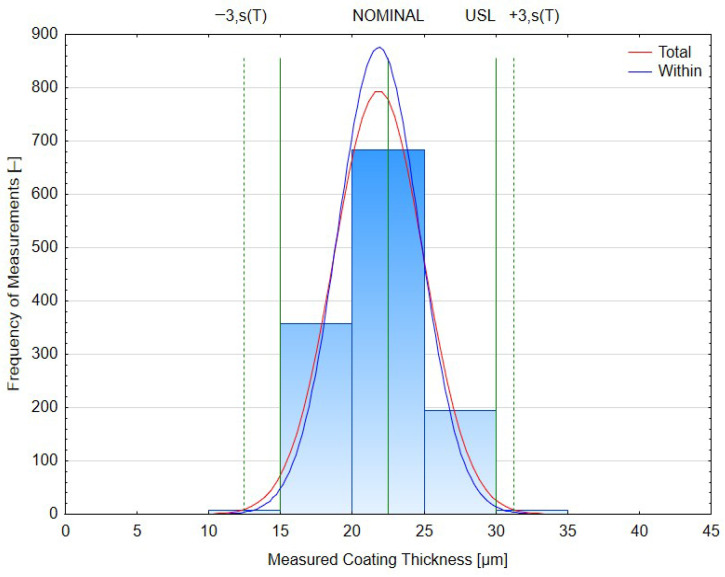
Histogram of Coating Thickness Distribution with Normal Fit.

The homogeneity of coating thickness among the 50 analyzed deposition stations was further assessed using a one-way ANOVA test. The obtained results revealed statistically significant differences between group means (F(49, 1200) = 3.49, *p* < 0.001). The between-group mean square (MS = 31.3) exceeded the within-group mean square (MS = 9.0), indicating that the inter-station variability was approximately 3.5 times greater than the random measurement variation within individual stations (Figure 4).

The magnitude of the station-related effect was further quantified using eta-squared (η^2^), defined as the ratio of between-group variance to total variance (5):

(5) $$\eta^{2}=\frac{SS_{\mathrm{between}}}{SS_{\mathrm{between}}+SS_{uncertainty}}$$

Substituting the obtained values gives (6):

(6) $$\eta^{2}=\frac{1534.2}{1534.2+10,762.2}=0.125$$

This indicates that approximately 12.5% of the total variability in coating thickness can be attributed to positional differences between deposition stations, while 87.5% is explained by random variation within stations. Such a value corresponds to a small-to-moderate effect size, confirming high process uniformity and reproducibility. In large-scale CED coating systems, such variations are expected due to the geometric configuration of the hanger, the positioning of samples relative to the anodes, and local fluctuations in bath conductivity or temperature. These factors influence the local throwing power of the bath and lead to thickness deviations typically below ±2 µm, which do not compromise either the functional or protective performance of the deposited film.

**Figure 5:**
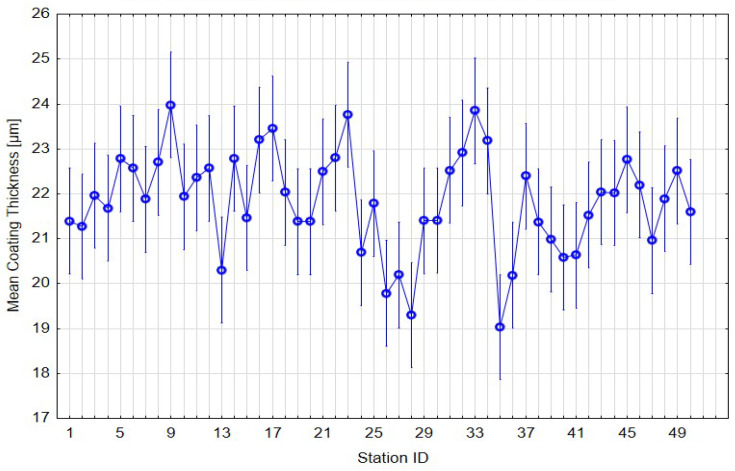
Mean coating thickness per station with 95% confidence intervals.

The graphical representation of mean coating thickness for each deposition station (Figure 5) indicates a stable process with low dispersion. The statistical evaluation (F(49, 1200) = 3.49, *p* < 0.001) (Table 6) revealed minor but measurable differences among stations. However, all means fell within the specification limits (15–30 µm), and the confidence intervals exhibited substantial overlap, confirming that these deviations are statistically but not technologically significant. Such local fluctuations are consistent with expected variations in cathodic electrodeposition, often caused by geometry-related shielding and electrolyte flow conditions.

**Figure 6:**
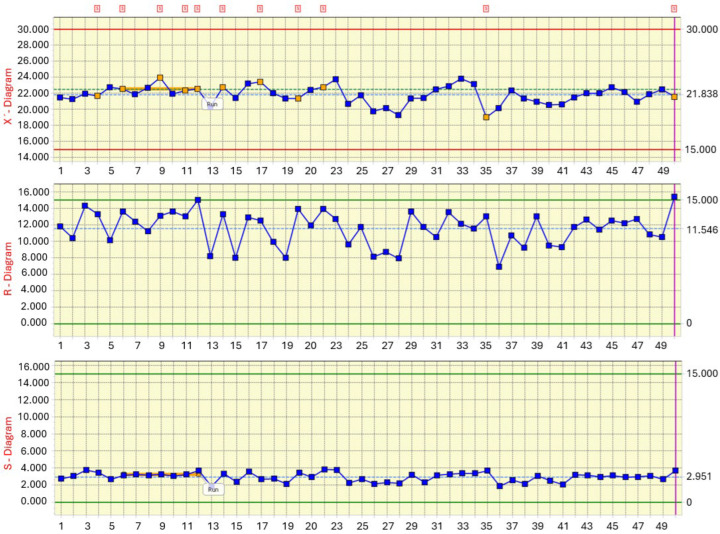
Shewhart X^¯^–R–S control charts for CED coating thickness across 50 deposition stations.

The stability of the electrodeposition process was further assessed using classical Shewhart control charts constructed from 50 subgroups, each representing one deposition station with 25 repeated thickness measurements. Figure 6 shows the combined X^¯^, R- and S-charts, which capture both the variation in station means and the within-station dispersion. The X^¯^-chart illustrates the mean coating thickness for each of the 50 stations. All subgroup means lie well inside the control limits, which essentially coincide with the technological specification window of 15–30 µm. The central line of the chart corresponds to the overall mean of 21.84 µm, in agreement with the descriptive statistics (Table 5). The profile of the X^¯^-chart exhibits only mild oscillations between approximately 20 and 24 µm, with a short run of points above the center line in the first third of the sequence, which is automatically flagged by the software. From the SPC perspective, the absence of points beyond the control limits and the lack of pronounced trends or cycles confirm that no dominant special cause affected the process. The observed pattern is consistent with the ANOVA result, where the between-station effect (η^2^ ≈ 0.125) was classified as small-to-moderate and technologically acceptable.

The R-chart captures the within-station range of thickness values. The average range is approximately 11.5 µm, with individual subgroup ranges fluctuating between 8 and 14 µm. All ranges remain clearly inside the control limits, indicating that the short-term dispersion within each station is stable and governed by common causes only. The shape of the R-chart supports the conclusion that the local variability originated from inherent process features—such as micro-scale bath turbulence, local current density gradients and part geometry—rather than from sporadic disturbances or measurement artefacts. The S-chart provides a more robust estimate of within-station variability in terms of standard deviation. The subgroup standard deviations cluster tightly around the central line at approximately 2.95 µm, which agrees well with the within-sample σ used in the capability analysis (≈2.85–2.95 µm). A short run of slightly elevated S-values is visible around stations 10–13, but the corresponding points remain far from the upper control limit and therefore do not indicate a loss of control. The overall stability of the S-chart confirms that the process dispersion is time-invariant and that the difference between total σ (3.14 µm) and within-station σ (≈2.9 µm) is mainly caused by systematic positional effects quantified previously by ANOVA.

**Figure 7:**
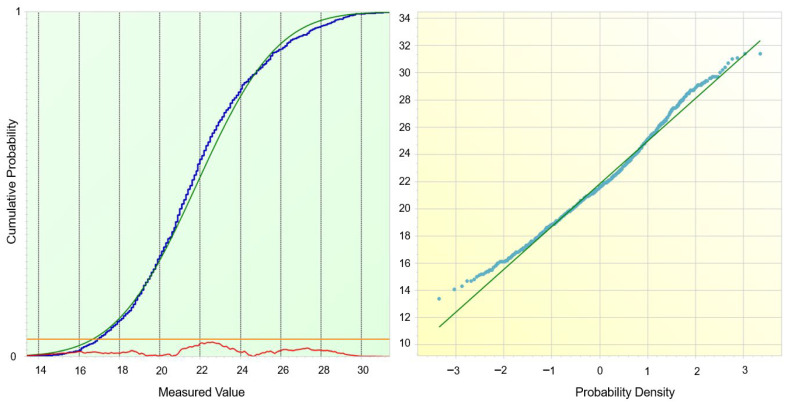
Cumulative Distribution Analysis and Normality Goodness-of-Fit Evaluation.

The empirical distribution of coating thickness was compared with the normal distribution using four complementary tests implemented in PalstatCAQ (Figure 7). All of them rejected the hypothesis of normality at the 5% significance level: χ^2^ test (*p* = 0.0209), Kolmogorov–Smirnov test (*p* = 0.00156), Shapiro–Wilk test (*p* ≈ 3.1 × 10^−9^) and Anderson–Darling test (*p* ≈ 2.8 × 10^−9^).

The cumulative distribution plot shows that the empirical curve follows the fitted normal distribution very closely in the central part of the range, while systematic deviations appear in both tails. This behaviour is confirmed by the Q–Q plot, where the majority of points align with the reference line, but slight curvature and spreading are visible for the lowest and highest quantiles. In combination with the earlier ANOVA results, these findings suggest that the global distribution is only approximately normal and that the observed deviations in the tails are caused mainly by the superposition of slightly different station-specific distributions (geometrical shielding, local conductivity and temperature effects) rather than by a fundamentally non-Gaussian mechanism.

**Figure 8:**
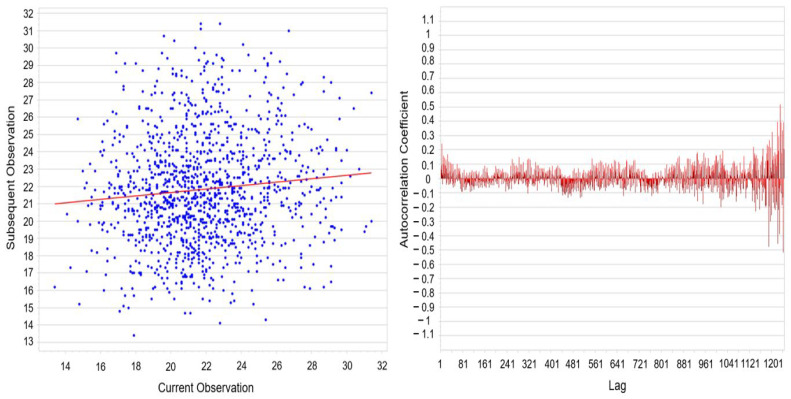
Autocorrelation Scatter Plot and Lag-Dependent Autocorrelation Function.

Overall, the quantitative evaluation confirms the absence of pronounced clustering in the measurement data and supports the assumption of approximate statistical independence between successive observations (Figure 8). This finding is critical for ensuring the validity of the capability analysis, as it requires unbiased estimation of both within-process and overall standard deviation. The observed variability is primarily attributable to moderate spatial heterogeneity among individual stations, while no indications of temporal drift or significant serial correlation were identified.

### 3.1. Process Capability and Performance Evaluation

To complement the analysis of variance across deposition stations, a comprehensive process capability evaluation was conducted to quantify the statistical robustness of the cathodic electrodeposition coating process. The assessment was performed using Statistica (TIBCO Software Inc., Palo Alto, CA, USA) with the Process Capability Analysis (Cp, Cpk and Pp, Ppk), Tolerance Intervals LSL (15.00 µm) and USL (30.00 µm), and Raw Data module (Table 7).

The obtained Cp = 0.878 indicates that the total process spread slightly exceeds the tolerance window, while Cpk = 0.808 confirms a small shift in the process mean toward the lower specification limit. The difference between Cp and Cpk (Δ = 0.07) is consistent with the non-centering factor K = 0.0884, representing an approximate 8.8% deviation from the nominal target. The Cp–Cpk difference therefore quantifies the practical impact of the mean shift: while the intrinsic spread of the process would be close to acceptable if it were perfectly centered, the current offset toward the lower specification limit reduces the effective capability observed at the customer interface. The capability ratio CR = 1.255 (inverse of Cp) implies that the process variation is approximately 25% larger than the specified tolerance width. This small offset is typical of large-scale cathodic electrodeposition systems, where local field gradients and hydrodynamic asymmetry result in non-uniform current density distribution.

The obtained Pp = 0.797 and Ppk = 0.726 show that the process remains statistically stable, but its long-term dispersion slightly increases compared to the short-term case (Cp = 0.878). This reduction can be attributed to positional differences among the 50 analyzed stations, previously confirmed by one-way ANOVA as a significant yet small factor (F(49, 1200) = 3.49, *p* < 0.001). The corresponding effect size η^2^≈12.5% quantifies the contribution of station-to-station variability to the overall process variance, explaining the observed Ppk decline by roughly 0.07 compared with Cp. The Z-bench values (2.06–2.11 σ) correspond to a process yield of approximately 93–94% within tolerance, which translates to 19 300 ppm of non-conforming parts. Although this level does not reach the “world-class” Six-Sigma threshold (3.0 σ ≈ 99.73%), it is typical for electrophoretic coating systems operating under industrial conditions with large hanging frames. The Taguchi capability index (Cpm = 0.78) confirms the presence of minor mean shift from the target value, yet the overall process variability remains limited, ensuring functional conformity of all parts. According to ISO 22514-2:2017 [44] and VDA 239-100 [5], processes with Cpk > 1.33 are classified as capable, while those in the range 0.67 < Cpk < 1.00 are conditionally capable. The present process, with Cpk = 0.81 and Ppk = 0.73, therefore falls within the conditionally capable category—statistically stable but with potential for improvement.

The difference between total and within-sample sigma values (3.14 µm vs. 2.85 µm) suggests that approximately 10–15% of the observed variability arises from geometric and positional effects within the coating bath, including electric field non-uniformity, part orientation, and electrolyte flow rate. Nevertheless, all measured values remain well within the functional range of 15–30 µm, ensuring uniform corrosion protection and aesthetic quality of the coated components. From a process engineering perspective, optimization of anode–cathode spacing, bath circulation, and hanger design could further reduce the process spread and shift the mean toward the nominal target, potentially increasing capability indices above Cp/Cpk = 1.00. Based on the same dataset and specification limits (LSL = 15 µm, USL = 30 µm, target = 22.5 µm), PalstatCAQ calculated capability and performance indices using several ISO 22514 [25,44]-compliant estimators of standard deviation (levels L1–L4). The summary is presented in Table 8. Across all four levels, the ratio Pp/Cp was approximately 0.80–0.89, while Ppk/Cpk remained close to 0.73–0.90, indicating that the long-term performance of the process is slightly worse than its short-term potential capability. These values are in very good agreement with the Statistica results (Cp = 0.878, Cpk = 0.808, Pp = 0.797, Ppk = 0.726), confirming the robustness of the capability assessment with respect to the chosen statistical software and sigma estimator.

### 3.2. Comparative Analysis of Capability Indices (ISO 22514)

In accordance with ISO 22514-2:2017 [44] and automotive practice, processes with Cpk > 1.33 are considered fully capable, whereas the interval 0.67 < Cpk < 1.00 corresponds to conditionally capable processes. The present electrodeposition line, with Cpk values ranging between 0.701 and 0.726 and Ppk values between 0.708 and 0.726 across the four evaluation levels (L1–L4), therefore falls consistently into the conditionally capable category. The global potential capability (Pp/Cp ≈ 0.796–0.870) indicates that the overall process spread is only moderately larger than the tolerance field, while the non-centering indices (Ppk/Cpk and Ppkl/Cpkl) reveal a slight negative shift in the mean toward the lower specification limit. Conversely, the upper-side indices (Ppku/Cpku ≈ 0.866–0.892) demonstrate that the upper tolerance margin is less critical, confirming that deviations are primarily concentrated below the nominal target. The PalstatCAQ analysis (Table 8) confirms the earlier Statistica-based conclusion: the process shows moderate inter-station variability, a slight mean offset, and ~6–7% of parts outside the 15–30 µm window. Control chart and autocorrelation results indicate temporal stability, with performance dominated by common causes and no evidence of strong serial correlation or drift.

### 3.3. Failure Mode and Effects Analysis (FMEA) of the Electrodeposition Coating Process

To complement the statistical evaluation of coating thickness, inter-station variability, and overall process capability, a comprehensive Failure Mode and Effects Analysis (FMEA) was conducted across the entire value stream of the coated electric charging station, in accordance with the AIAG–VDA methodology [45]. Critical process steps—including surface inspection, part hanging, chemical degreasing, cathodic electrodeposition, curing, packaging, assembly, and shipment—were systematically analyzed regarding their functional roles, potential failure modes, underlying causes, and resultant effects on the final product quality.

Within the material receipt and surface inspection stages, principal failure modes encompassed incorrect order identification and inadequate surface preparation. These were assigned Severity (S) scores of 7, Occurrence (O) of 4, and Detection (D) of 1. Human uncertainty was identified as the dominant root cause, mitigated through operator retraining and multi-tiered inspection procedures. Compliance with material specifications for the subsequent KTL process was verified for each delivery, ensuring uninterrupted process continuity. During part hanging, potential failure modes included incorrect part placement and uncoated contact points, with S = 7, O = 3–3, and D = 3. Root causes were primarily procedural non-compliance and improper fixture positioning. Countermeasures encompassed detailed work instructions, targeted operator training, and staged inspections. The chemical degreasing stage was highlighted as a critical control point. Insufficient bath temperatures could lead to non-uniform coating layers (S = 7, O = 3, D = 3). Preventive measures incorporated continuous monitoring of degreasing bath conditions and meticulous process documentation to maintain conformity with specifications. The cathodic electrodeposition process presented multiple potential failure modes, including insufficient curing (S = 7, O = 3, D = 3), inadequate deposition time (S = 7, O = 3, D = 3), and incorrect input of line parameters (S = 7, O = 4, D = 2). The root causes were predominantly associated with suboptimal curing conditions, improper process parameters, and operator uncertainty. Mitigation strategies involved rigorous control of the curing program, visual and tactile inspection of deposited layers, adherence to standard operating procedures, and operator retraining.

Post-deposition quality checks further identified risks related to layer appearance, parameter compliance, and thickness measurement (S = 5, O = 3–4, D = 2–5), which were addressed via routine calibration, standardized procedures, and inspection protocols. Packaging and assembly/shipment phases were associated with failure modes such as misplacement of components and damage during handling (S = 7, O = 3–4, D = 1–3). Identified root causes included human uncertainty and procedural lapses.

### 3.4. Risk Reduction and Process Enhancement of the Electrodeposition Coating Process

Following the FMEA assessment, targeted optimization measures were implemented to reduce the risk priority numbers (RPNs) of the identified critical failure modes (FM1–FM8). Table 9 summarizes the initial RPN values, expected reduction percentages, and the projected post-optimization RPN for each failure mode.

**Figure 9:**
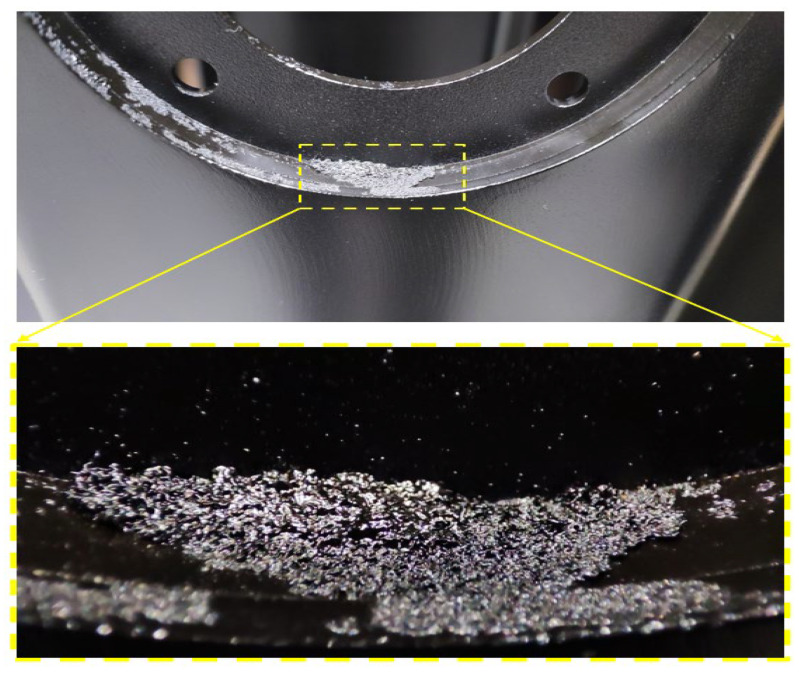
Representative surface defect evidencing FM1/FM3.

Figure 9 illustrates a representative defect observed on the inner circular flange of the coated component. The rough agglomerated structure indicates incomplete surface activation and local field shielding, consistent with the high-risk failure modes FM1 (inadequate degreasing/surface contamination) and FM3 (incorrect hanging).

This defect is directly aligned with the mechanisms identified through the integrated statistical assessment. The presence of a locally thick, granular, and poorly coalesced layer corresponds to the same variability sources highlighted by ANOVA, where station-dependent differences (η^2^ ≈ 12.5%) indicated geometric shielding and inconsistent surface activation as contributors to non-uniform film build-up. From the SPC perspective, these localized surface instabilities represent the microstructural expression of the small deviations captured in the X^¯^–R–S charts, which remained within control limits yet exhibited mild oscillations reflecting positional effects. The observed defect also provides a physical explanation for the moderate capability values obtained in the Cp/Cpk (0.878/0.808) and Pp/Ppk (0.797/0.726) indices: while the global process remains statistically controlled, local interruption of wetting or electrochemical deposition—precisely as seen in this region—creates extreme values in the lower and upper distribution tails, thereby reducing long-term capability. The convergence between the visual defect morphology and the highest-risk failure modes in the FMEA (FM1 and FM3, RPN before = 252–270) confirms that inadequate degreasing and improper hanging are not isolated issues but structurally embedded drivers of the observed statistical behaviour. This photographic evidence therefore supports the FMEA risk ranking, strengthening the causal linkage between failure modes and measured performance.

The spatial thickness variability and conditional process capability observed in this study can be mechanistically attributed to surface-defect phenomena previously quantified by the authors under controlled laboratory conditions on the same cathodic electrodeposition line. The study by Peti et al. [23] demonstrated that deviations in polymerization and curing conditions induce insufficient crosslinking, residual solvent retention, and internal stress development, which subsequently manifest as microcracking, localized delamination, and reduced coating integrity. These defect mechanisms were experimentally shown to degrade adhesion performance (ISO 2409 [46], Grade 0–1), impact resistance (ISO 6272-1) [47], and the structural continuity of the CED layer, despite nominal coating thickness remaining within specification limits. The capability indices obtained in the present industrial-scale study (Cp = 0.878, Cpk = 0.808, Pp = 0.797, Ppk = 0.726) can be directly interpreted in the context of these previously published laboratory-scale investigations conducted on the same cathodic electrodeposition line. In that study, statistically designed experiments focusing on polymerization parameters yielded potential capability indices exceeding Cp > 1.8 and Pp ≈ 1.4 under optimized curing conditions, whereas performance indices remained substantially lower (Cpk < 0.5, Ppk < 0.4) due to systematic mean shifts and distributional asymmetry. These deviations were directly correlated with curing-induced microcracking and localized delamination, which were confirmed by SEM–EDX analysis and accompanied by measurable degradation of adhesion and impact resistance. Similarly, the reported study [20] demonstrated that, under controlled deposition voltages of 250–300 V and optimized degreasing temperatures (40–50 °C), average coating thickness values ranged between 17.7 and 22.0 μm with improved compositional homogeneity confirmed by EDX mapping. Stable carbon (~49 wt.%), oxygen (~6–7 wt.%), and silicon-rich additive distributions were observed across the 20–25 μm coating profile. In contrast, non-optimized pretreatment and deposition conditions resulted in reduced thickness uniformity (as low as 12.2–14.8 μm) and increased spatial inconsistency, despite nominal specification compliance.

### 3.5. Prediction Model of Layer Thickness—Implementation of Machine Learning

The predictive performance of the coating thickness model was assessed through a systematic comparison between experimentally measured thickness values, model-predicted outputs, and the corresponding residuals, as summarized in Table 10.

The evaluation is based on the complete industrial dataset comprising 1250 measurements, organized according to the deposition station identifier, with each station represented by a consistent set of 25 experimentally acquired thickness values. The predictive framework was intentionally constructed using exclusively station-resolved thickness data, without incorporating additional process parameters, to preserve the direct linkage between the measured output and the spatially dependent behaviour of the deposition system. This data-driven structure enables the model to capture station-specific deposition characteristics and systematic spatial variability inherent to the industrial process. The close correspondence between the mean measured and predicted thickness values, together with a residual mean value approaching zero, demonstrates the absence of systematic prediction bias and quantitatively demonstrates that the model reliably captures the dominant process trends governing coating thickness evolution under real production conditions.

**Figure 10:**
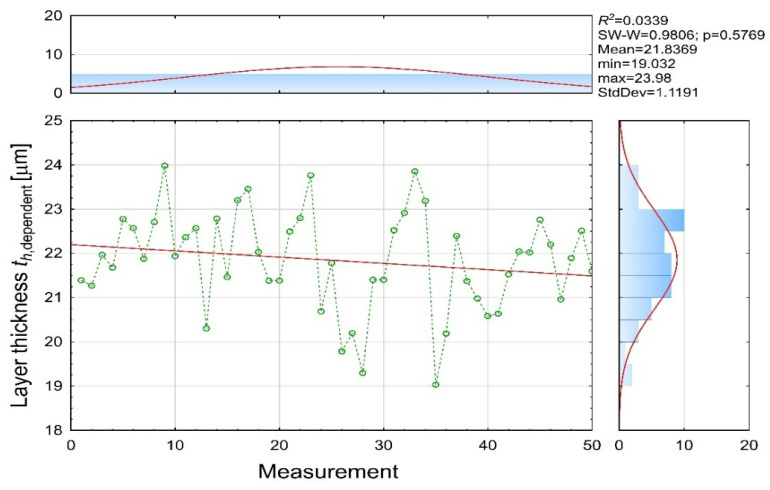
Experimental coating thickness [µm] (th dependent variable).

The measured coating thickness values (Figure 10) exhibit noticeable variability, ranging from 19.03 to 23.98 μm, with a mean of 21.84 μm. The dataset follows a normal distribution (SW-W = 0.9806; *p* = 0.5769), and the observed fluctuations reflect natural instabilities commonly present in electrodeposition systems, such as localized changes in bath conductivity, variations in component geometry, temperature non-uniformities, and differences in wet-film formation. The slightly negative slope of the regression line confirms the absence of a dominant long-term drift within the 50-measurement sequence.

**Figure 11:**
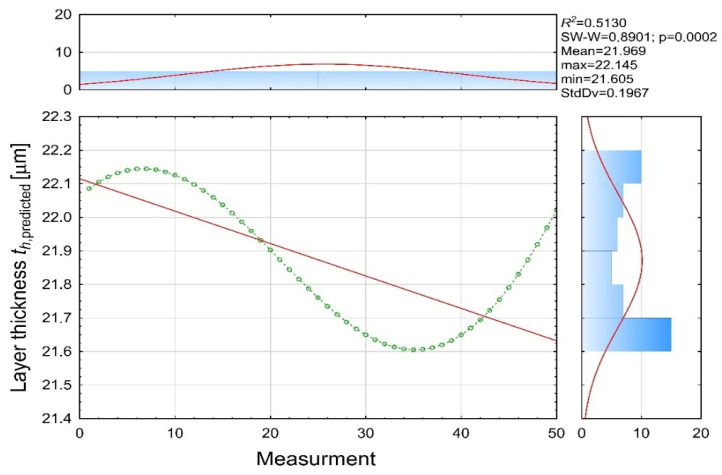
Model-predicted coating thickness [µm] (th predicted variable).

The predicted values (Figure 11) show a significantly reduced standard deviation (Std.Dev = 0.1967 μm) and remain within a narrow interval (21.61–22.15 μm) with a mean of 21.97 μm. This confirms that the model captures the overall deposition trend but does not replicate the high-frequency fluctuations present in the real process. The linear regression of predicted values (R^2^ = 0.5130) indicates that approximately half of the variability is explained by the regression structure, which is consistent with a model derived from a partially nonlinear dataset.

**Figure 12:**
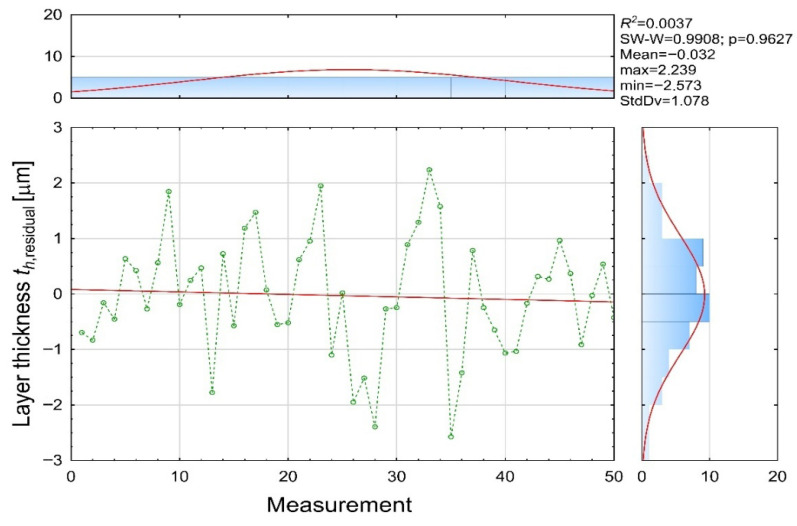
Residual [µm] (th residual variable).

Residuals (Figure 12) were calculated as the difference between the dependent and predicted coating thickness values. Their distribution indicates almost zero systematic bias and demonstrates that residuals are purely random and independent of measurement order of an electric-charging station.

These findings correspond with the numerical values, that small deviations (|r| < 0.5 μm) dominate most measurements (e.g., Variables 1, 4, 10, 11, 18, 25, 48), medium deviations (0.5–1.5 μm) occur occasionally (e.g., Variables 7, 13, 15, 20, 27, 36) and larger deviations (>2 μm) appear in outlier cases (e.g., Variables 28 and 35), typically associated with known process instabilities such as insufficient wetting or local voltage imbalances.

**Figure 13:**
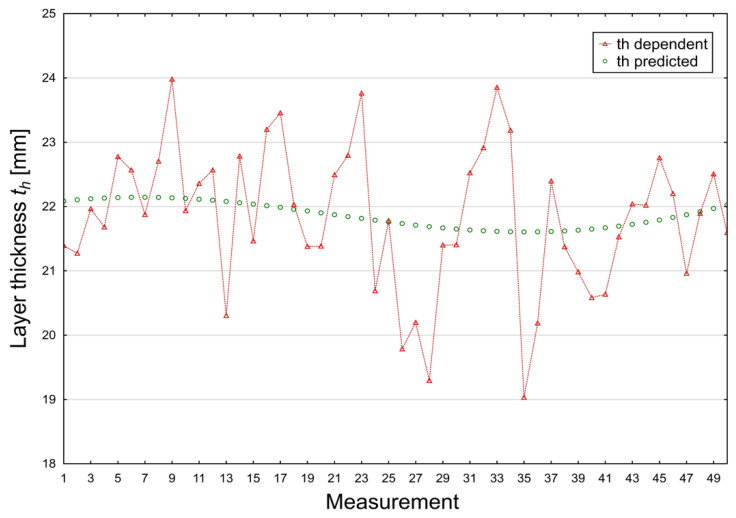
Comparison Plot Between Dependent and Predicted Thickness [µm].

The comparison between experimentally measured and model-predicted thickness values (Figure 13) highlights the inherent distinction between real process behaviour and the abstracted structure of the predictive model. The dependent experimental values (red markers) exhibit pronounced local variability, spanning approximately 19–24 μm, which reflects short-term instabilities and micro-scale heterogeneities characteristic of cathodic electrodeposition processes. Despite these fluctuations, the predictive model accurately reproduces the overall trend of coating thickness evolution while effectively suppressing process-related noise.

This behaviour demonstrates the model’s capability to capture dominant deposition patterns rather than transient local disturbances. As a result, the model is well suited for monitoring thickness stability, detecting systematic deviations, and supporting predictive quality control in industrial electrodeposition operations. The combined evaluation of experimental values and predicted outputs and residual distributions quantitatively demonstrates that the model provides a robust representation of the global deposition behaviour, even in the presence of natural short-term variability. Larger deviations are observed only at extreme thickness values and can be attributed to known instability mechanisms inherent to electrodeposition systems. For most observations, the residuals remain small, approximately normally distributed, and unbiased.

## 4. Conclusions

This study addresses a significant methodological gap in the evaluation of cathodic electrodeposition (CED) coatings by delivering an integrated, industrial-scale assessment of spatial variability, statistical stability, process capability, and risk-oriented quality. Unlike predominantly laboratory-based investigations focused on parameter optimization, the present work is based on 1250 coating-thickness measurements collected across 50 deposition stations, enabling robust characterization of both local and global process behaviour under real production conditions.

From a technical and methodological perspective, the principal findings can be summarized as follows:

- The CED process was demonstrated to operate in a statistically stable and controlled regime, with all measured thickness values remaining within the 15–30 μm specification limits, as confirmed by SPC and autocorrelation analysis.
- One-way ANOVA revealed statistically significant inter-station differences (F(49, 1200) = 3.49, *p* < 0.001); however, the calculated effect size (η^2^ ≈ 12.5%) indicates that positional effects contribute only a moderate portion of total variability, while approximately 87.5% originates from common-cause within variation.
- Shewhart X^¯^–R–S control charts confirmed long-term process stability, with subgroup means and dispersions consistently remaining within control limits and no evidence of special-cause variation. The within-station standard deviation clustered around 2.9 μm, supporting time-invariant dispersion behaviour.
- Distributional analysis using χ^2^, Kolmogorov–Smirnov, Shapiro–Wilk, and Anderson–Darling tests identified minor deviations from normality primarily confined to distribution tails. These deviations were attributed to the superposition of slightly heterogeneous station-specific distributions rather than to a fundamentally non-Gaussian deposition mechanism.
- Process capability analysis classified the system as conditionally capable, yielding short-term indices Cp = 0.878 and Cpk = 0.808, and long-term performance indices Pp = 0.797 and Ppk = 0.726. The observed Cp–Cpk difference (Δ ≈ 0.07) and non-centering factor K = 0.088 quantify a moderate mean shift toward the lower specification limit, indicating that capability limitations arise from systematic geometric and positional effects rather than random process noise.
- AIAG–VDA FMEA identified surface contamination, bath chemistry instability, and improper hanging configuration as the dominant process risk drivers, with initial RPN values ranging from 252 to 336. Targeted corrective actions resulted in RPN reductions of 50–62.5%.
- A machine-learning-based predictive model, developed exclusively from station-resolved thickness data, accurately reproduced the macroscopic deposition trend with negligible bias (mean residual −0.03 μm).

By integrating spatial ANOVA, SPC, ISO 22514 [25,44]-compliant capability analysis, AIAG–VDA FMEA, and data-driven prediction within a unified framework, this study provides an industrially validated methodology that quantitatively links station-resolved variability, statistical stability, and process capability with underlying technological risk factors. The resulting approach enables reliable identification of dominant sources of thickness variability, objective prioritization of corrective actions, and transparent assessment of long-term production capability in complex cathodic electrodeposition lines.

Future research should extend the present findings through durability-oriented investigations, particularly Neutral Salt Spray (NSS) testing and cyclic corrosion exposure, to rigorously evaluate long-term functional performance. While microstructural characterization has been comprehensively addressed in previous studies, subsequent work should prioritize mechanical and operational loading tests. In the specifically cited parallel investigations, coating thickness was determined according to ISO 2808:2019 [27] to accurately quantify spatial variability and ensure uniform deposition; adhesion was assessed via the cross-cut method following ISO 2409:2020 [46] to evaluate interfacial bonding strength and coating integrity; and dynamic mechanical resistance was assessed using a falling-weight impact apparatus in accordance with ISO 6272-1:2011 [47] to characterize the coating’s ability to withstand mechanical loading and impact-induced stress. Collectively, these methodologies provide a rigorous framework for linking material properties with functional robustness, enabling quantification of how spatial thickness variability and curing-induced stress gradients influence coating performance and service reliability under operational conditions.

## Figures and Tables

**Table 1:** Chemical composition of S235JRC+N structural steel (mass. %) [26].

Element	C [%]	Mn [%]	P [%]	S [%]	N [%]	Cu [%]	Si [%]
Content	≤0.17	≤1.40	≤0.035	≤0.035	≤0.012	≤0.55	≤0.03

**Table 2:** Mechanical properties of S235JRC+N structural steel [26].

Thickness [mm]	Yield Strength Re [MPa]	Tensile Strength Rm [MPa]	Elongation A [%]	Testing Temperature [°C]	Impact Energy KV [J]
≤16	≥235	360–510	≥26	20	≥27
16–40	≥225	350–500	≥24	20	≥27
40–63	≥215	340–490	≥22	20	≥27

**Table 3:** Operational Conditions of Cathodic Electrodeposition and Pre-Treatment Steps.

Process Step	Duration [min]	Temperature [°C]	Voltage [V]	Ultrafiltrate
Chemical Degreasing	3–8	65–75	–	–
Phosphating	5:00	45–55	–	–
Cathodic Electrodeposition	5:00	30–35	280	–
Ultrafiltrate Rinse	0:20	–	–	Yes
Curing	30:00	190–210	–	–

**Table 4:** Established capability index thresholds for evaluation of coating process performance.

Index	Required Value	Interpretation
Cp, Cpk	≥1.33	Process considered capable and statistically stable
Pp, Ppk	≥1.50	Process considered robust and long-term-capable

**Table 5:** Descriptive Statistics of Coating Thickness Measurements [µm].

SUM of Station	Valid N	Mean Thickness [µm]	Min [µm]	Max [µm]	Variance [µm^2^]	Std.Dev [µm]	Standard Uncertainty [µm]	Kurtosis [-]
50	1250	21.84	13.40	31.40	8.97	3.14	0.59	–0.04

**Table 6:** ANOVA summary for station variability.

Parameter	Symbol	Value
Sum of Squares	SS	1534.2 [µm^2^]
Degrees of Freedom	df	49
Mean Square	MS	31.31 [µm^2^]
F-statistic	F	3.491
Significance	*p*	<0.001

**Table 7:** Process capability and performance indices for cathodic electrodeposition thickness measurements.

Potential Capability Index	Cp	0.8780	Short-term capability assuming process centering
Actual Capability Index	Cpk	0.8084	Short-term capability considering process shift
Potential Performance Index	Pp	0.7968	Long-term process capability
Actual Performance Index	Ppk	0.7263	Long-term capability considering centering deviation
Capability Ratio	CR	1.2550	Ratio of process spread to tolerance width
Non-centering Correction Factor	K	0.0884	Quantifies the mean offset from the nominal target
Taguchi Capability Index	Cpm	0.7796	Potential capability considering deviation from target
Z-benchmark (potential)	Zbench (pot.)	2.11 σ	Ideal sigma level assuming perfect centering
Z-benchmark (overall) **	Zbench (total)	2.06 σ	Actual sigma level based on real process spread
Out-of-spec parts per million	PPM Total	19,305.96 ppm	Estimated non-conforming units
Lower Process Capability	PPL	0.7263	Lower-side capability
Upper Process Capability	PPU	0.8672	Upper-side capability

**—Actual sigma level based on real process spread.

**Table 8:** Comparison of capability and performance indices according to ISO 22514 [44] for four evaluation levels (L1–L4).

L	Index	X (99.865–0.135%) Fit	X (99.865–0.135%) Real	$\sqrt{\left(S^{2}\right)}$	s′/c_4_	R′/d_2_	σ
L1—X′	Pp/Cp	0.796	0.885	0.834	0.838	0.851	0.796
	Ppk/Cpk	0.726	0.872	0.760	0.764	0.776	0.726
	Ppkl/Cpkl	0.726	0.900	0.760	0.764	0.776	0.726
	Ppku/Cpku	0.866	0.872	0.907	0.912	0.926	0.866
L2—$\tilde{X}$	Pp/Cp	0.796	0.885	0.834	0.838	0.851	0.796
	Ppk/Cpk	0.719	0.876	0.734	0.738	0.749	0.701
	Ppkl/Cpkl	0.719	0.896	0.734	0.738	0.749	0.708
	Ppku/Cpku	0.870	0.876	0.934	0.939	0.953	0.892
L3—X″	Pp/Cp	0.796	0.885	0.834	0.838	0.851	0.796
	Ppk/Cpk	0.726	0.872	0.760	0.764	0.776	0.726
	Ppkl/Cpkl	0.726	0.872	0.760	0.764	0.776	0.726
	Ppku/Cpku	0.866	0.872	0.907	0.912	0.926	0.866
L4—${\tilde{X}}^{\prime }$	Pp/Cp	0.796	0.885	0.834	0.838	0.851	0.796
	Ppk/Cpk	0.721	0.875	0.741	0.745	0.757	0.708
	Ppkl/Cpkl	0.721	0.875	0.741	0.745	0.757	0.708
	Ppku/Cpku	0.869	0.875	0.927	0.932	0.946	0.885

**Table 9:** RPN Values Before and After Optimization.

Failure Mode (FM)	Description	S	O	D	RPN Before	Expected Reduction (%)	RPN After
FM1	Inadequate degreasing/surface contamination	9	6	5	270	55%	122
FM2	Incorrect bath chemistry (pH, conductivity)	8	7	6	336	62.5%	126
FM3	Incorrect hanging/uneven paint layer	7	6	6	252	60%	101
FM4	Insufficient inspection/incomplete documentation	8	6	7	336	50%	168
FM5	Low degreasing bath temperature	7	5	6	210	55%	95
FM6	Incorrect curing parameters	8	5	5	200	50%	100
FM7	Damage during loading/human uncertainty	7	6	5	210	55%	95
FM8	Visual non-conformities after cataphoresis	7	5	6	210	55%	95

**Table 10:** Comparison of Experimental and Predicted Thickness Values with Residual Analysis.

SUM of Variable (Station ID)	Valid N	th Dependent Mean Value [µm]	th Predicted Mean Value [µm]	Residual Mean Value [µm]
50	1250	21.84	21.97	−0.03

## Data Availability

The original contributions presented in this study are included in the article/Supplementary Material. Further inquiries can be directed to the corresponding author.

## Supplementary Materials

The following supporting information can be downloaded at: https://www.mdpi.com/article/10.3390/ma19020330/s1, Table S1: Descriptive Statistics of Coating Thickness Measurements; Table S2: Comparison of Experimental and Predicted Thickness Values with Residual Analysis.
