# Supplementary material for: Process Capability Assessment and Surface Quality Monitoring in Cathodic Electrodeposition of S235JRC+N Electric-Charging Station
Source: Materials (Basel). 2026 Jan 14;19(2):330. doi: 10.3390/ma19020330 (PMC12842731; doi:10.3390/ma19020330)
Supplement: Supplementary file 1 [file materials-19-00330-s001.zip › materials-4063007-supplementary.pdf]

Table S1. Descriptive Statistics of Coating Thickness Measurements

Station ID	Valid N	Mean	Min	Max	Variance	Std.Dev	Standard Error	Kurtosis
1	25	21,39200	16,40000	28,20000	6,68493	2,585524	0,517105	0,77226
2	25	21,27200	16,00000	26,40000	9,29293	3,048431	0,609686	-0,82956
3	25	21,96400	15,00000	29,30000	14,11073	3,756426	0,751285	0,09676
4	25	21,68000	14,30000	27,60000	12,07000	3,474191	0,694838	-0,45102
5	25	22,77600	17,30000	27,40000	7,19523	2,682393	0,536479	-0,55786
6	25	22,56800	17,10000	30,70000	9,92977	3,151153	0,630231	0,47102
7	25	21,87600	16,60000	29,00000	10,53440	3,245674	0,649135	-0,22199
8	25	22,70400	17,90000	29,10000	10,04207	3,168922	0,633784	-0,77174
9	25	23,98000	17,90000	31,00000	10,62333	3,259346	0,651869	-0,30413
10	25	21,93600	15,10000	28,70000	9,33240	3,054898	0,610980	0,95798
11	25	22,36000	14,10000	27,10000	10,48667	3,238312	0,647662	0,18725
12	25	22,56800	14,70000	29,70000	13,78643	3,713009	0,742602	0,22832
13	25	20,30400	15,80000	24,00000	3,41540	1,848080	0,369616	0,62524
14	25	22,78400	16,90000	30,20000	11,24473	3,353317	0,670663	-0,17959
15	25	21,46400	17,10000	25,10000	5,61240	2,369050	0,473810	-0,88059
16	25	23,20000	16,70000	29,60000	12,71917	3,566394	0,713279	-0,77940
17	25	23,45600	18,90000	31,40000	7,27923	2,698005	0,539601	1,87415
18	25	22,03200	18,10000	28,00000	7,48477	2,735830	0,547166	-0,34394
19	25	21,38000	16,80000	24,80000	4,61250	2,147673	0,429535	-0,50471
20	25	21,38400	16,50000	30,40000	12,03973	3,469832	0,693966	0,58542
21	25	22,49200	16,60000	28,50000	8,53743	2,921889	0,584378	-0,32648
22	25	22,79600	17,50000	31,40000	14,78873	3,845612	0,769122	-0,53696
23	25	23,76400	17,30000	30,00000	14,34157	3,787026	0,757405	-1,06521
24	25	20,68800	16,70000	26,30000	5,16527	2,272722	0,454544	0,94291
25	25	21,78000	16,90000	28,60000	7,34583	2,710320	0,542064	0,30516
26	25	19,78400	15,30000	23,40000	4,68307	2,164039	0,432808	-0,60082
27	25	20,19600	16,80000	25,50000	5,30040	2,302260	0,460452	-0,20797
28	25	19,29600	15,50000	23,40000	4,95373	2,225698	0,445140	-0,90840
29	25	21,40000	16,10000	29,70000	10,33167	3,214291	0,642858	0,40461
30	25	21,40400	16,20000	27,90000	5,40623	2,325131	0,465026	1,92026
31	25	22,52400	18,30000	28,80000	9,99273	3,161128	0,632226	-0,48811
32	25	22,91200	16,20000	29,70000	10,84527	3,293215	0,658643	-0,39145
33	25	23,85200	17,50000	29,60000	11,40010	3,376403	0,675281	-0,98574
34	25	23,18400	18,20000	29,70000	11,30223	3,361879	0,672376	-0,56655
35	25	19,03200	13,40000	26,40000	13,52810	3,678057	0,735611	-0,94020
36	25	20,18800	17,00000	23,90000	3,52027	1,876237	0,375247	-0,95535
37	25	22,39600	17,80000	28,50000	6,72707	2,593659	0,518732	0,07168
38	25	21,37200	16,10000	25,30000	4,69877	2,167664	0,433533	0,25197
39	25	20,98400	15,70000	28,70000	9,54973	3,090264	0,618053	0,10684
40	25	20,58400	15,90000	25,40000	6,33973	2,517883	0,503577	-0,47154
41	25	20,63600	15,50000	24,80000	4,32907	2,080641	0,416128	1,15520
42	25	21,52800	16,00000	27,70000	10,31960	3,212413	0,642483	-0,31567
43	25	22,04000	16,50000	29,10000	10,03083	3,167149	0,633430	0,29546
44	25	22,02000	17,30000	28,70000	8,82750	2,971111	0,594222	0,02454
45	25	22,75600	16,90000	29,40000	9,67340	3,110209	0,622042	-0,13886
46	25	22,20000	15,40000	27,60000	8,55833	2,925463	0,585093	0,34142
47	25	20,96000	16,40000	29,10000	8,88833	2,981331	0,596266	0,89637
48	25	21,89200	17,60000	28,40000	9,60743	3,099586	0,619917	-0,43472
49	25	22,50800	18,80000	29,30000	7,18160	2,679851	0,535970	0,17025
50	25	21,59600	15,70000	31,10000	13,75373	3,708603	0,741721	0,45575
**SUM**	**1250**	**21,83688**	**13,40000**	**31,40000**	**8,96849**	**3,137672**	**0,58955**	**–0,04035**

Table S2. Comparison of Experimental and Predicted Thickness Values with Residual Analysis

Variable	th dependent	th predicted	Residual
**1**	21,39200	22,08565	-0,69365
**2**	21,27200	22,10491	-0,83291
**3**	21,96400	22,12036	-0,15636
**4**	21,68000	22,13202	-0,45202
**5**	22,77600	22,13991	0,63609
**6**	22,56800	22,14410	0,42390
**7**	21,87600	22,14468	-0,26868
**8**	22,70400	22,14174	0,56226
**9**	23,98000	22,13542	1,84458
**10**	21,93600	22,12589	-0,18989
**11**	22,36000	22,11332	0,24668
**12**	22,56800	22,09791	0,47009
**13**	20,30400	22,07988	-1,77588
**14**	22,78400	22,05948	0,72452
**15**	21,46400	22,03696	-0,57296
**16**	23,20000	22,01260	1,18740
**17**	23,45600	21,98668	1,46932
**18**	22,03200	21,95951	0,07249
**19**	21,38000	21,93138	-0,55138
**20**	21,38400	21,90261	-0,51861
**21**	22,49200	21,87353	0,61847
**22**	22,79600	21,84446	0,95154
**23**	23,76400	21,81572	1,94828
**24**	20,68800	21,78762	-1,09962
**25**	21,78000	21,76050	0,01950
**26**	19,78400	21,73466	-1,95066
**27**	20,19600	21,71040	-1,51440
**28**	19,29600	21,68801	-2,39201
**29**	21,40000	21,66778	-0,26778
**30**	21,40400	21,64997	-0,24597
**31**	22,52400	21,63482	0,88918
**32**	22,91200	21,62257	1,28943
**33**	23,85200	21,61343	2,23857
**34**	23,18400	21,60758	1,57642
**35**	19,03200	21,60519	-2,57319
**36**	20,18800	21,60641	-1,41841
**37**	22,39600	21,61136	0,78464
**38**	21,37200	21,62012	-0,24812
**39**	20,98400	21,63276	-0,64876
**40**	20,58400	21,64934	-1,06534
**41**	20,63600	21,66986	-1,03386
**42**	21,52800	21,69431	-0,16631
**43**	22,04000	21,72266	0,31734
**44**	22,02000	21,75484	0,26516
**45**	22,75600	21,79077	0,96523
**46**	22,20000	21,83035	0,36965
**47**	20,96000	21,87343	-0,91343
**48**	21,89200	21,91986	-0,02786
**49**	22,50800	21,96946	0,53854
**50**	21,59600	22,02205	-0,42605
